# No Stone Left Unturned: Bouveret Syndrome Treated With Electrohydraulic Lithotripsy and Open Extraction With Pyloric Exclusion and Gastrojejunostomy

**DOI:** 10.7759/cureus.39661

**Published:** 2023-05-29

**Authors:** Kaiser F Kabir, John P Hanna, Hossein Haghbin

**Affiliations:** 1 Internal Medicine, Michigan State University College of Osteopathic Medicine, East Lansing, USA; 2 Internal Medicine, Ascension Macomb-Oakland Hospital, Warren, USA; 3 Gastroenterology, Ascension Providence Hospital, Southfield, USA

**Keywords:** bouveret syndrome, electrohydraulic lithotripsy, choleduodenal fistula, upper endoscopy, advanced endoscopy, gallstone extraction, bouveret's syndrome

## Abstract

Bouveret syndrome is ectopic gallstone impaction and obstruction of the duodenum or pylorus affecting a small minority of gallstone ileus cases. There have been advances in its endoscopic management, but this remains a challenging condition to treat successfully. We present a patient with Bouveret syndrome who required open surgical extraction and gastrojejunostomy after attempts of endoscopic retrieval and electrohydraulic lithotripsy (EHL). A 79-year-old man with a medical history of gastroesophageal reflux disease, chronic obstructive pulmonary disease on 5 liters of oxygen at baseline, and coronary artery disease with recent stenting presented to the hospital with three days of abdominal pain and vomiting. CT of the abdomen/pelvis demonstrated gastric outlet obstruction, a 4.5 cm gallstone in the proximal duodenum, cholecystoduodenal fistula, gallbladder wall thickening, and pneumobilia. Esophagogastroduodenoscopy (EGD) demonstrated a black pigmented stone impacted in the duodenal bulb with ulceration of the inferior wall. Repeated Roth net retrieval attempts of the stone were unsuccessful even after biopsy forceps were used to trim the stone’s margins. The next day, EGD with EHL used 20 shocks of 200 watts, allowing for partial stone removal and fragmentation, but the majority of the stone remained stuck to the wall. Laparoscopic cholecystectomy was attempted but was converted to an open extraction of the gallstone from the duodenum, pyloric exclusion, and gastrojejunostomy. The gallbladder remained in place, and the cholecystoduodenal fistula was not surgically repaired. The patient experienced significant postoperative pulmonary insufficiency and remained on the ventilator with failure of multiple spontaneous breathing trials. Postoperative imaging showed resolution of pneumobilia but a small amount of contrast leaked from the duodenum revealing the fistula's persistence. After 14 days of unsuccessful ventilator weaning, the family opted for palliative extubation. Advanced endoscopic techniques have been regarded as the first-line intervention for Bouveret syndrome as there is low morbidity and mortality associated with them. However, there is a reduced success rate compared to surgical intervention. Open surgical management has high morbidity and mortality in the elderly and comorbid patients commonly affected by this condition. Thus, the risks and benefits must be weighed and individualized for each patient with Bouveret syndrome before therapeutic intervention.

## Introduction

Bouveret syndrome describes the rare presentation of an impacted gallstone in the duodenum or the pylorus causing obstruction, which is reported in less than 3% of gallstone ileus cases [1]. First described by Leon Bouveret in 1896, there have been significant advances in treatment options for this condition, including advanced endoscopic techniques [2]. We present a case of an elderly patient with Bouveret syndrome complicated with cholecystoduodenal fistula, suspected cholecystitis, and pneumobilia, who, after endoscopic retrieval attempts and electrohydraulic lithotripsy (EHL), required open surgical removal of the stone from the duodenum followed by pyloric exclusion and gastrojejunostomy.

## Case presentation

**Figure 1:**
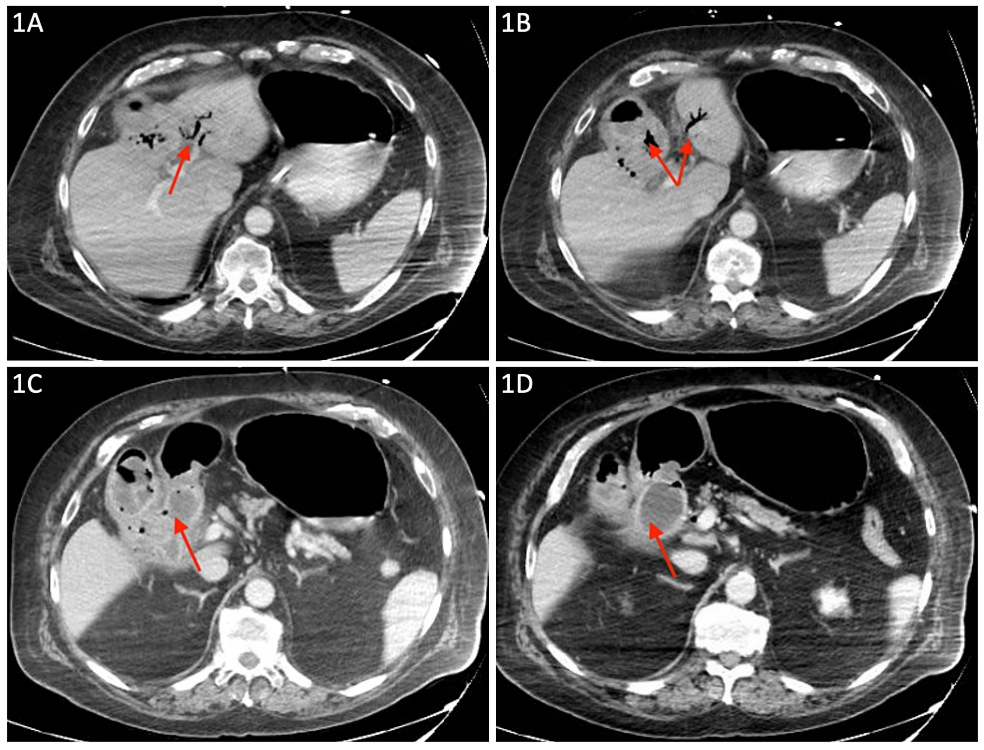
CT of the abdomen/pelvis Pneumobilia is seen in figure (A), with accompanying air in the gallbladder in figure (B). A gallstone in the duodenum and cholecystoduodenal fistula are seen in figure (C), with the full gallstone visualized in the proximal duodenum in figure (D).

A 79-year-old man with a medical history of gastroesophageal reflux disease, chronic obstructive pulmonary disease (COPD) on 5 liters nasal cannula at baseline, and coronary artery disease with percutaneous intervention to multiple vessels and stent placement five months ago, on aspirin and clopidogrel, presented to the hospital with abdominal pain and vomiting. He had been hospitalized four months earlier for community-acquired pneumonia and COPD exacerbation requiring bilevel positive airway pressure (BiPAP) and discharged after three days with home health care. Abdominal pain began three days ago and was non-specific and dull. He had multiple episodes of non-bloody, non-bilious vomiting. On physical examination, the abdomen was distended, tender in the upper quadrants, and without peritoneal signs. He required increased oxygen with 6 liters via nasal cannula but otherwise, his vitals were stable. His initial relevant labs included leukocytosis of 14.29. CT of the abdomen/pelvis with contrast demonstrated gastric outlet obstruction with a radiolucent 4.5 cm gallstone in the proximal duodenum, cholecystoduodenal fistula, gallbladder wall thickening, and pneumobilia (Figure 1). The patient's echocardiogram revealed an ejection fraction of 60-65%, no systolic or diastolic dysfunction, severely calcified mitral valve leaflets with moderate to severe stenosis without significant regurgitation, and mild tricuspid regurgitation.

**Figure 2:**
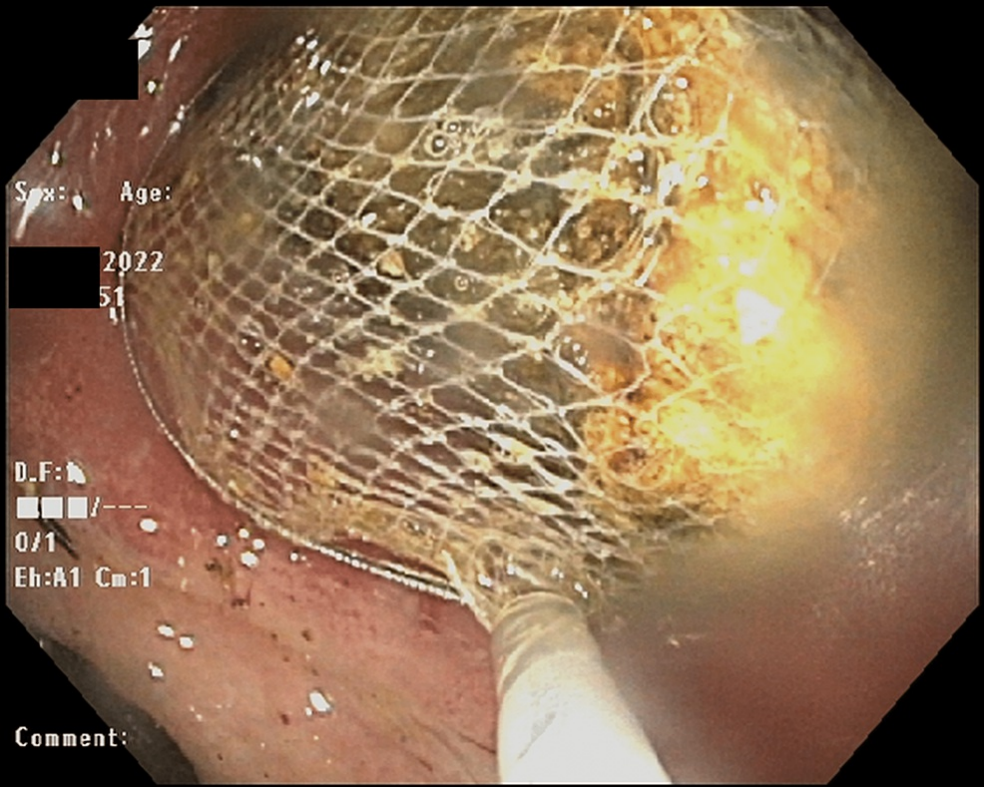
Esophagogastroduodenoscopy with Roth net retrieval attempt

He underwent esophagogastroduodenoscopy (EGD) on the third day of hospitalization that demonstrated stomach distension, and a large black pigmented stone impacted at the duodenal bulb with ulceration of the duodenum's inferior wall from the stone fragments. Roth net retrieval was first attempted but was unsuccessful. Biopsy forceps were then utilized to trim the margins of the stone with subsequent retrievement failure (Figure 2).

**Figure 3:**
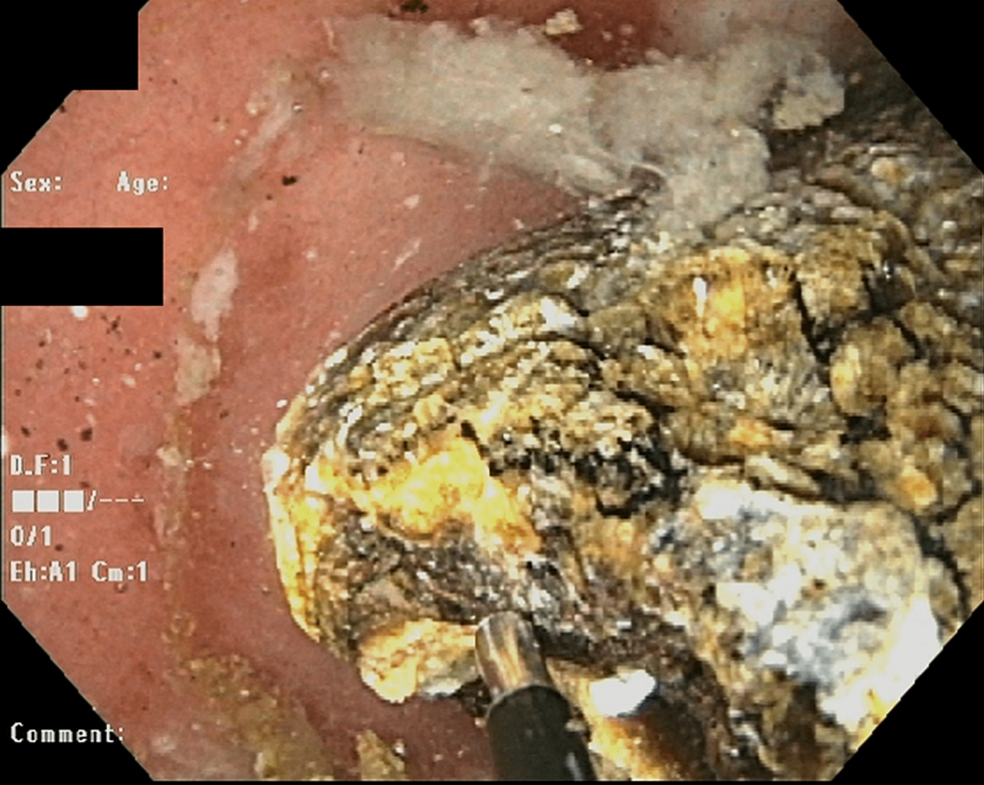
Fragmented stone after electrohydraulic lithotripsy

On the next day, EGD with EHL used 20 shocks of 200 watts, allowing stone fragmentation with partial removal (Figure 3). Despite this, the majority of the stone remained stuck to the duodenal bulb apex. As this second endoscopic approach for the stone removal was unsuccessful, general surgery took the patient to the operating room (OR). Preoperative laboratory results are listed in Table 1.

**Table 1:** Preoperative laboratory values

Lab test	Value	Reference value
White blood cell count	14.66 K/mcL	4.0-11.0 K/mcL
Hemoglobin	12.4 g/dL	13.5-17.5 g/dL
Hematocrit	39.40%	41-53%
Mean corpuscular volume	93 fL	80-100 fL
Platelets	213k/mcL	150-400k/mcL
International normalized ratio	1.46	-
Aspartate aminotransferase	18 unit/L	0-45 unit/L
Alanine aminotransferase	17 unit/L	0-45 unit/L
Alkaline phosphatase	59 iUnits/L	20-130 iUnits/L
Total bilirubin	0.3 mg/dL	0-1.5 mg/dL
Direct bilirubin	<0.2 mg/dL	0-0.4 mg/dL
Albumin	2.3 gm/dL	3.5-5.0 gm/dL
Lactic acid	1.6 mmol/L	0.5-2.0 mmol/L
Blood urea nitrogen	25 mg/dL	8-20 mg/dL
Serum creatinine	0.61 mg/dL	0.7-1.5 mg/dL
Sodium	138 mmol/L	135-145 mmol/L
Potassium	3.5 mmol/L	3.5-5.4 mmol/L
Chloride	99 mmol/L	98-109 mmol/L
Bicarbonate	31 mmol/L	23-34 mmol/L
Calcium	8.6 mg/dL	8.4-10.5 mg/dL
Magnesium	1.7 mEq/L	1.3 to 1.9 mEq/L
Phosphorus	3.1 mg/dL	2.4-4.5 mg/dL

First, a robotic laparoscopic cholecystectomy was attempted, which was converted to an open approach because the stone could not be removed laparoscopically due to its size and firmness. The open approach allowed for manual extraction of the gallstone from the proximal duodenum. A purse-string suture was performed closing the duodenal mucosa, followed by pyloric exclusion and gastrojejunostomy. The gallbladder was not removed, and the fistula was left to close on its own. Intravenous ceftriaxone, metronidazole, and fluconazole were started postoperatively. The patient remained intubated. While in the ICU, the patient was found to have acute deep vein thrombosis (DVT) of the right subclavian and axillary veins. He was started on an intravenous unfractionated heparin infusion. A repeat CT on postoperative day three demonstrated less air in the gallbladder and resolution of pneumobilia. On postoperative day five, an upper gastrointestinal gastrografin study was administered via a nasogastric tube and demonstrated a small amount of leakage into the gallbladder fossa, representing the cholecystoduodenal fistula's presence. The patient experienced significant postoperative respiratory insufficiency with escalating ventilator settings, at first, followed by multiple failed spontaneous breathing trials. His postoperative ICU course was further complicated by fevers attributed to his DVT, as he exhibited no other signs of infection, as well as elevated troponins in the setting of normal electrocardiograms, indicative of demand ischemia. He remained on intravenous fluid support and total parenteral nutrition since the nature of his surgery precluded the usage of gastric or jejunal feeding tubes. After 14 days of unsuccessful ventilator weaning and with consideration of his quality of life, the patient's durable power of attorney, alongside family members, agreed on a comfort care approach with palliative extubation. The patient passed away within hours.

## Discussion

This case demonstrates many of the common findings for a rare condition. Rigler’s triad, which encompasses an ectopic gallstone, pneumobilia, and small bowel obstruction, is reported to be pathognomonic for Bouveret syndrome [3]. A comprehensive review of cases reported only 24% of patients presented with a visible cholecystoduodenal fistula in CT, and gallstones are visualized during EGD in about 69% of the reported cases [4]. The repair of gallstone obstruction in the gastric pylorus or duodenum can only be accomplished via endoscopic or surgical techniques [5]. Advanced endoscopy's minimally invasive nature mitigates the complications of this condition [1,5]. Successful use of only Roth net retrieval without dependence on EHL, laser lithotripsy, or surgery has been reported [3]. As there is very low morbidity and mortality associated with endoscopic retrieval, some sources report a reduced success rate in comparison to surgical enterolithotomy or gastrostomy, either on its own or coupled with cholecystectomy and fistula repair [1,4,5]. However, open surgical management is associated with increased morbidity and mortality, especially as this condition often affects elderly patients over the age of 65 years of age [2,6]. Although fistula repair cannot be completed with endoscopy, it is oftentimes unnecessary to repair immediately because the fistula may close on its own [2]. Thus, as seen in this case, despite the presence of cholecystoduodenal fistula on imaging, upper endoscopy was performed first, and even after open surgical intervention, it was not repaired after the manual extraction of the stone. EHL was attempted in this case, with partial success, in combination with Roth net retrieval. EHL is typically useful in the fragmentation of stones in the pancreatic and biliary ducts, but its successful use with water immersion in Bouveret syndrome has been reported. The water immersion reduces the risk of damage to the surrounding duodenal or gastric mucosa [2]. Recently, a case report by Watanabe et al. described using EHL to create a narrow hole within a large obstructing stone and subsequent balloon expansion of the hole split the stone in half to relieve the gastric outlet obstruction [7]. Bouveret syndrome with concomitant choledocholithiasis has been successfully treated with endoscopic stone removal, endoscopic retrograde cholangiopancreatography (ERCP), and subsequent cholecystectomy [6]. In our case, during the open approach, removal of the stone was possible, and the pyloric exclusion was performed to prevent gastric contents from entering the duodenum. Some studies have found that pyloric exclusion confers longer hospitalizations and increased mortality, but this technique may be used when large gallstones must be extracted from the proximal duodenum in Bouveret syndrome [8,9]. The gallbladder was not removed in this case. The utilization of cholecystectomy has been debated in Bouveret syndrome [2,4,5]. The advantage of removing the gallbladder in a one-stage surgery reduces the risks of subsequent cholecystitis, cholangitis, and gallbladder malignancy [2,5]. While early identification of this syndrome was accomplished and earnest attempts were made using EHL, the patient still required surgical treatment, and his significant comorbidities predisposed him to postoperative complications. The postoperative clinical course also prevented him from returning to the OR for subsequent cholecystectomy and fistula closure. However, it is questionable that performing a cholecystectomy and definitive fistula closure would have changed the eventual outcome. This case brings to the forefront the difficult decisions that are made in developing a therapeutic intervention strategy and managing its complications. The patient's clinical course underscores the importance of both developing and improving accessibility to advanced endoscopic techniques, with reliance on surgery only if necessary.

## Conclusions

Bouveret syndrome is a rare condition encompassing an ectopic gallstone with small bowel obstruction and air in the biliary tree, also known as pneumobilia. Its early recognition and diagnosis are absolutely crucial, as this condition can be difficult to manage, due to its need for endoscopic and/or surgical intervention. The typical patient is usually in the seventh decade or above, with multiple comorbidities, and thus increased risks are associated with its therapies. Advanced endoscopic techniques are proposed as a first-line treatment due to their minimally invasive nature. Upper endoscopy incorporating EHL, Roth net retrieval, and laser lithotripsy can be used by the skilled endoscopist. Utilization and access to such techniques are crucial, as it is associated with lower morbidity and mortality in Bouveret syndrome. However, surgical intervention will still be necessary in complicated cases. Ultimately, an individualized and interprofessional patient approach is critical when developing a treatment strategy to minimize risk, with full consideration of the complications that this condition and its interventions may have on the patient's life.
